# Physicochemical Characterization of Exudate Gum from *Neltuma flexuosa* (ex *Prosopis flexuosa*)

**DOI:** 10.3390/polym18151850

**Published:** 2026-07-28

**Authors:** Alba Benuzzi, Franco Tonelli, Gisela Melo, Mauricio Filippa, Martin Masuelli

**Affiliations:** 1 Área de Química Física, Departamento de Química, Facultad de Química, Bioquímica y Farmacia, Universidad Nacional de San Luis, Ejército de los Andes 950, San Luis ZC 5700, Argentina or gmmelo@unsl.edu.ar (G.M.); or mauricio.filippa@gmail.com (M.F.); 2 Laboratorio de Ingeniería de las Reacciones Químicas, FICA-UNSL, Ruta 148 Extremo Norte, Villa Mercedes, San Luis ZC 5730, Argentina; ftonelli@unsl.edu.ar; 3 Instituto de Física Aplicada (INFAP-CONICET-UNSL), Área de Química Física, Departamento de Química, Facultad de Química, Bioquímica y Farmacia, Universidad Nacional de San Luis, Ejército de los Andes 950, San Luis ZC 5700, Argentina

**Keywords:** *Neltuma flexuosa*, exudate, gum, physicochemical characterization, molecular weight, solution permittivity

## Abstract

*Neltuma flexuosa* exudate gum (NFEG) is a complex polysaccharide and a promising sustainable substitute for arabic gum, boasting an exceptional 92% extraction yield. Proximate analysis reveals an arabinogalactan–protein structure (89.9% carbohydrates, 3.2% protein) essential for its superior emulsifying and stabilizing properties. Composed mainly of galactose and arabinose, its random coil morphology ensures low viscosity at high concentrations, ideal for beverages. The presence of uronic acids (11.6%) provides a strong polyelectrolytic character, enhancing solubility and surface activity. Physicochemically, it mirrors arabic gum with an intrinsic viscosity of 19.90 cm^3^/g and thermal stability up to 237.4 °C. Structural analysis via FTIR and XRD confirms its amorphous polysaccharide nature and high chain flexibility. Furthermore, NFEG exhibits intrinsic bioactivity, including antioxidant properties confirmed by DPPH and reducing power assays. These dual functional properties present to NFEG as a high-value industrial hydrocolloid.

## 1. Introduction

*Neltuma flexuosa* (ex *Prosopis flexuosa*) is a key tree species in arid and semi-arid regions of South America, especially Argentina. It is known for its adaptation to drought and its ecological and cultural value. One of its notable characteristics is the production of an exudate gum [1,2,3,4,5].

The exudate of *Neltuma flexuosa*, also known as “algarroba dulce”, is produced through wounds on the trunk or main branches of the tree, similar to mesquite gum [6]. This gum is water-soluble and have properties that make them comparable to arabic gum, a product widely used in various industries [7].

Mesquite gum, also known as “chúcata” in some regions, is a gummy exudate produced by the mesquite tree (genus Neltuma ex Prosopis) as a defense mechanism under stressful conditions, such as extreme heat or lack of water. This polysaccharide is a protein arabinogalactan whose physicochemical and functional properties are very similar to those of gum arabic, a product with wide range of uses in the food, pharmaceutical, and beverage industries [6]. Due to the scarcity and supply problems of arabic gum, mesquite gum has gained interest as a viable alternative. Its main applications include stabilizing and emulsifying, and encapsulating flavors and aromas. It is used in dressings, sauces, mayonnaise, and to extend the shelf life of fruits and vegetables. In traditional uses include being used as a natural sweetener, antiseptic, and analgesic, in cosmetics, and in traditional medicine. Several species of the Neltuma genus produce mesquite gum, but the most studied and cited for their production are: *Neltuma laevigata*, *Neltuma juliflora*, *Neltuma glandulosa* and *Neltuma velutina* [6,7,8,9].

While specific information on the detailed chemical composition of *Neltuma flexuosa* exudate may be limited in the available literature, it is known that plant gums from species of the genus *Neltuma* (ex *Prosopis*) are generally complex polysaccharides. These are mainly composed of monosaccharides such as arabinose, galactose, rhamnose and glucuronic acid, and present a branched structure [10,11,12,13]. The functional properties of this gum make it a hydrocolloid with thickening and stabilizing capacity [14]. The exudate of *Neltuma flexuosa* represents a natural resource with significant potential, although its study and use are still under development compared to other natural gums [15].

The *Neltuma flexuosa* exudate gum possesses similar properties to mesquite gum [7,16,17] and gum arabic (produced by species of the genus Acacia) [18,19]. This similarity is crucial, since arabic gum is a food additive and industrial ingredient highly valued for its thickening, stabilizing and emulsifying abilities. This comparison suggests a similar application potential for locust bean gum in the food, pharmaceutical and other industries [20,21,22,23,24].

The hydrocolloid nature of *Neltuma flexuosa* exudate, which thickens and stabilizes, makes it an interesting candidate for use in the food industry. Research has explored its application in products such as soups and desserts, showing acceptable sensory characteristics and no syneresis (phase separation), indicating its viability as a food additive. This aligns with the revaluation of *Neltuma* spp. fruits as forgotten and underutilized species, whose promotion can positively impact the conservation of native forests and regional economies [14,25,26].

Optimizing the extraction process for these edible gums, such as those from *Neltuma flexuosa*, is an active area of research. This is essential to maximize their yield and quality, allowing for more efficient and sustainable use of this resource [27,28,29].

Despite its potential, the large-scale use of *Neltuma flexuosa* exudate gum can face challenges related to collection, processing, and standardization of its properties, factors that are key to its acceptance in industrial markets. However, its native origin in arid and semi-arid regions, where the plant is a dominant and resilient species, opens up opportunities for the development of local value chains that benefit rural communities and promote sustainable management practices [30,31,32,33,34].

Gums are widely used as food additives as thickeners and gelling agents. The *Neltuma* family (*Neltuma alpataco* and *Neltuma flexuosa*) is a source of gums, especially their seeds, in which galactomannans have been characterized. The objective of this work was to optimize the extraction conditions of gums from *N. flexuosa* and *N. alpataco* fruits, prioritizing yield and purity. Water extractions were performed using whole fruit flour, seed pieces, or seed meal. The dispersions were heated at 60 °C for 1 h and then centrifuged at 20,000× *g*. The resulting pellet was used for successive extractions. Two solvents were tested to precipitate gums from the supernatant: isopropanol or 96% ethanol. A gradient between 20 and 90% of the selected alcohol was tested. The gums were isolated using two techniques: filtration and centrifugation. The samples were dried in a convection oven at 60 °C. The percentage yield on a dry basis (gum mass/initial flour mass) was calculated. Purity was estimated by quantifying proteins using the Kjeldahl method (factor 6.25). The seed pieces (five extractions) had a yield of 13% while, in the first extraction, the whole fruit flour had a yield between 5% and 10%. Since the seed flour had a yield greater than 20%, the tests were continued with this flour. Ethanol precipitated a greater mass of gums (30%) than isopropanol (20%) in the first extraction. The amount of protein in the precipitates was independent of the number of extractions and the solvent chosen. The gradient showed that 90% of the mass precipitates at a concentration of 30% vol. of ethanol. There was no difference in yield between filtration and centrifugation techniques to separate gums; centrifugation was chosen because it is faster. The concentration of proteins in gums decreased to less than 15% of the total precipitate when centrifugation was applied at 20,000× *g*. No significant differences in extraction yield were observed between both species. In conclusion, the best yields and purity in the extraction of gums from *N. alpataco* and *N. flexuosa* fruits were achieved with aqueous extraction from seed flour, separation by centrifugation at 20,000× *g*, precipitation of gums from the supernatant with 30% vol. ethanol, and centrifugation to recover the pellet [15].

Nutritional and social determinants, and potential impact of food use of carob trees (*Neltuma* spp. ex *Prosopis*) in urban and neo-rural populations of Córdoba. (Reference to the revaluation of Neltuma species). The study and valorization of *Neltuma flexuosa* exudate not only opens doors to new industrial applications, but also contributes to the conservation of a tree species vital to arid ecosystems and to the local communities that have traditionally depended on it. The sweet carob tree, now scientifically known as *Neltuma* spp., is an emblematic tree of arid and semi-arid South America, especially in Argentina. This species, vital for its capacity to adapt to drought and its ecological and cultural value, is recognized for the production of a gummy exudate that oozes from wounds on its trunk or main branches [31,32,33].

This exudate, commonly called “algarroba dulce”, is a water-soluble hydrocolloid with properties comparable to those of the renowned arabic gum [35]. While the specific chemical composition of *Neltuma flexuosa exudate* gum requires further research, it is known that the gums of species of the genus *Neltuma* are mostly complex polysaccharides, composed of sugars such as arabinose, galactose, rhamnose, and glucuronic acid, which give them a branched structure and functional properties as thickeners and stabilizers [36]. Its potential lies not only in its industrial application, but also in its key role in the sustainable development of regional economies and the conservation of native forests [15].

### Physicochemical Properties of Neltuma flexuosa Exudate Gum

Preliminary studies on purified *Neltuma flexuosa* exudate gum (NFEG) have revealed the following properties: at 27 °C, the intrinsic viscosity is 20.79 cm^3^/g. This measurement is comparable to that of arabic gum (19.81 cm^3^/g), suggesting a similar behavior in solution. In solution, NFEG tends to acquire a spherical shape, with an ν_a/b_ axis ratio of 2.5 and a specific volume of 8.32 cm^3^/g. The intrinsic viscosity of NFEG increases with temperature. The flexibility of the biopolymer is 0.2251 K^−1^, indicating a very high flexibility of the NFEG chain [32].

As already mentioned, *Neltuma flexuosa* exudate is a water-soluble gum composed primarily of complex polysaccharides. These polysaccharides are of the arabinogalactan type, which are usually branched and contain units of arabinose, galactose, rhamnose, and uronic acids (such as glucuronic acid) [37,38,39,40,41].

Below are some specific properties that have been reported in the literature, often compared to arabic gum due to their functional similarities: *Neltuma flexuosa* (ex *Prosopis flexuosa*) gum is characterized as a polysaccharide with a high proportion of galactose and arabinose [4,32]. The presence of rhamnose and glucuronic acid has also been reported [32]. The proportion and type of these monosaccharides directly influence the properties of the gum. Tree exudate gums often contain a small protein fraction covalently associated with the polysaccharide structure, forming a glycoprotein [4,32]. This protein fraction is crucial for its emulsifying and stabilizing properties. Although exact values may vary depending on purification and source, the presence of protein is a distinctive feature. *Neltuma flexuosa* gum forms viscous solutions even at low concentrations [32,41]. Its intrinsic viscosity has been observed to be comparable to that of arabic gum, indicating similar behavior in solution [32]. Solutions of this gum can exhibit pseudoplastic (shear-thinning) behavior at higher concentrations, meaning that its viscosity decreases with increasing shear force. This is a desirable property for many applications in the food and pharmaceutical industries [32,41]. The viscosity of gum solutions has been reported to be relatively stable over a range of pH [41]. However, temperature can influence viscosity, generally decreasing it at higher temperatures [32]. The exudate is highly water-soluble, allowing it to form stable solutions in water. Its ability to hydrate and form viscous solutions is critical to its function as a thickener and stabilizer [32]. Exudate gums are typically light in color, ranging from light amber to colorless, depending on purity and collection conditions [42]. These are standard parameters for gum characterization and may vary slightly, but are typically low in purified products [42].

The physicochemical properties of *Neltuma flexuosa* exudated gum, particularly its polysaccharide composition, protein fraction, and rheological characteristics, make it comparable to arabic gum (*Acacia senegal* or *Acacia seyal*). This similarity is key because arabic gum is a hydrocolloid widely used in the food (as an emulsifier, stabilizer, and coating agent), pharmaceutical, cosmetic, and other industries [30,32]. The study of *Neltuma flexuosa* exudate gum is of great importance for: 1—Promoting the sustainable use of native species from arid areas; 2—offering an alternative to imported gums, which could reduce costs and promote local self-sufficiency; 3—its use is foreseen in the production of food (thickeners, stabilizers in desserts, sauces, beverages), pharmaceutical products (coating agents, binders in tablets) and cosmetics (emulsifiers), 4—despite its potential, research and standardization of the collection and purification processes are essential for its large-scale commercialization [30].

The objective is to evaluate Neltuma flexuosa exudate gum as a sustainable substitute for arabic gum using proximal analysis, acid hydrolysis, and characterization via FTIR, XRD, DSC and TGA-DTGA. The study aims to determine efficiency through a high-yield extraction process and validate physicochemical properties by measuring intrinsic viscosity, thermal stability, and chain morphology. Finally, it seeks to quantify bioactive and antioxidant capacity using DPPH and reducing power assays to confirm its industrial potential.

## 2. Materials and Methods

### 2.1. Raw Material

*Neltuma flexuosa* exudate gum (NFEG) was collected in “campus UNSL, Av. Ejército de los Andes 950, San Luis, Argentina”; the tree was located at −33.29′15.17″ S, −66.34′01.05″ W.

The procedure consisted of dissolving the exudate in distilled water and filtering it. The filtrate was precipitated with ethanol at a 70/30 ratio in distilled water and filtering again. The retentate is dried at 60 °C for 24 h [43]. The dried precipitate is crushed and ground into a fine powder, then packaged in a recipient with a lid.

The yield was calculated from 100 g of NFEG using the following equation:

(1) $$Yield\%=100\frac{{NFEG}_{sol}}{NFEG}$$

where NFEG is the initial mass (100 g) and NFEG_sol_ is the mass of the resulting filtration processes [44].

#### 2.1.1. Proximal Analysis

The sample underwent pulverization into a fine consistency following conventional procedures, and then it was subjected to a basic compositional breakdown (AOAC, 2012 [45]). The protein content was ascertained via the Kjeldahl–Arnold–Gunning technique. Subsequently, the aggregate protein quantity was computed by employing a conversion coefficient of 6.25 (AOAC. 920.12). Additionally, the protein output was computed and documented as milligrams of protein generated per liter of sample per hour. Total lipids were measured using the Soxhlet gravimetric protocol, which involved petroleum ether (Sigma Aldrich, St. Louis, MA, USA) (AOAC 945.39). Concurrently, raw fiber content was established through the sample digestion approach utilizing H_2_SO_4_ and NaOH (Sigma Aldrich) (AOAC 962.09). To determine the ash content, the sample was incinerated, and the weight discrepancy was utilized (AOAC 945.46). The water content (moisture) was derived by heating the material under a partial vacuum (AOAC 945.46). Carbohydrates were derived indirectly using the subsequent equation: Total Carbohydrates (%) = 100 − (Proteins + Total Lipids + Moisture + Ash). Adhering to these standardized methodologies guaranteed the precision and uniformity of our examination, which yields insightful data for prospective investigation or utilization across diverse sectors [45,46].

#### 2.1.2. Sugar Composition

The hydrolysis of NFEG was carried out at 100 °C under reflux with 1.7 M HCl for 2 h. After cooling, the remaining HCl was volatilized at 60 °C for 1 h until reaching a pH 6, approximately. The sugar composition was determined using glucuronic acid, methoxyglucuronic acid, galactose, rhamnose, and arabinose standards (all reagents from Sigma Aldrich, Darmstadt, Germany). The sugar composition was quantified by HPLC with a refractive index detector (Gilson™, Lewis Center, OH, USA) using a SweetSep™ AEX200 column (Antec Scientific, Alphen a/d Rijn, The Netherlands); the mobile phase had a flow rate of 1 mL/min in 20 mM NaOH modified with 10 mM NaOAc to reduce the retention of carbohydrates [47].

#### 2.1.3. Antioxidant Activity Assays

##### Reducing Power

The analysis of the reductive capacity inherent in polysaccharide solutions, documented in citation [48], required a sequence of processes. This technique called for exact quantification and controlled thermal exposure (incubation) to guarantee the generation of dependable data. The incorporation of chemical agents, specifically a phosphate buffering system (2 M, pH 6.6), potassium hexacyanoferrate (II) (1% wt.), trichloroacetic acid (2.5 mL, 10% wt.), and ferric chloride (0.2 mL), was paramount. These additions played a critical role in both terminating the chemical process and subsequently determining the light absorption (absorbance) at 700 nm. To validate the consistency of the results, the entire protocol was executed three times (in triplicate). Gallic acid functioned as the benchmark substance (positive control) for relative comparison. The final readings were presented in units of meq/mL of gallic acid (All reactive were Sigma Aldrich, St. Louis, MA, USA). This careful and detailed methodology enabled a comprehensive appraisal of the polysaccharides’ reducing strength, thereby highlighting the necessity for meticulousness in scientific procedures.

##### DPPH Scavenging Activity

The radical scavenging capacity against DPPH was evaluated using a modified procedure derived from source [49]. This assessment entailed combining 0.5 mL of the polysaccharide preparation (0.1 to 1 mL concentration) with a 0.15 M DPPH in 90% ethanol/water. Following a half-hour resting period (incubation) in the dark, the light absorption (absorbance) was measured at 517 nm. The overall antioxidant activity was subsequently calculated as a percentage, with results ultimately expressed as meq/g of gallic acid. All chemical agents utilized in this determination were procured from Sigma Aldrich (St. Louis, MA, USA).

##### Total Polyphenol Content

Total phenolic compounds were quantified utilizing a modified Folin–Ciocalteu assay as previously documented [48,50]. The procedure began by meticulously mixing a 5 mL sample volume with 1.5 mL of 2 N Folin–Ciocalteu reagent and 15 mL of 15% Na_2_CO_3_ solution in a volumetric flask, which was then filled to a final volume of 25 mL with distilled water. This resulting solution was subsequently kept away from light at ambient temperature for a two-hour reaction period. Following this incubation, the absorbance was measured at 760 nm. To guarantee accuracy and reliable quantification, a standard curve was prepared using gallic acid (GA) across six concentrations spanning 1 to 6 mg/L. The result is expressed in meq/g of gallic acid. The entire protocol was executed in duplicate to ensure reproducibility and precision of the results.

### 2.2. Fourier Transform Infrared Spectroscopy

In this study, Fourier Transform Infrared spectroscopy (FTIR), a common technique in analytical chemistry for identifying chemical compounds, was used. The spectra were obtained using a Nicolet PROTEGE 460 spectrometer (Madison, WI, USA) in two modes: diffuse reflectance (DRIFTS) and transmission. Data collection was performed in the 700–400 cm^−1^ range by scanning each sample 64 times to ensure accurate results. The combined use of both modes allowed for a detailed analysis of the chemical composition, structure, and molecular vibrations of the samples, ensuring the robustness and reliability of the obtained spectra for meaningful interpretation [50,51].

### 2.3. X-Ray Diffraction

X-ray diffraction (XRD) studies were performed using an advanced Rigaku ULTIMA IV type II instrument (Tokyo, Japan). This instrument employed a Cu Kα lamp with a wavelength (λ_B_) of 1.54 Å and a nickel filter for accurate measurements. Scanning was performed in the 2θ range from 3° to 60°, operating the equipment at 30 kV and 20 mA. Data collection was performed continuously at a speed of 3° per minute and a reading step of 0.02° [52].

### 2.4. Differential Scanning Calorimetry

Differential scanning calorimetry (DSC) was performed using an STA 449F3-Jupiter (Selb, Germany). Approximately 5 mg of the biopolymer sample was placed in an alumina crucible and heated from 25 °C to 400 °C at a constant rate of 5 °C/min. This process was carried out under a dynamic nitrogen atmosphere with a flow rate of 25 mL/min, which was selected to accurately analyze the thermal behavior, characteristic transitions, and stability of the biopolymer [53,54].

### 2.5. Thermogravimetric Analysis and Differential Thermogravimetric Analysis

The thermal stability of the polysaccharide films was determined by thermogravimetric analysis (TGA) using a TA Instruments TG 295 analyzer (New Castle, DE, USA). Samples of approximately 8 mg were heated at 10 °C/min under a pre-filtered N_2_ (99.99%) atmosphere with a flow rate of 50 mL/min using empty aluminum crucibles as references. Temperature calibration was performed using indium (melting point 156.60 °C) and the Curie point of Ni (353 °C) [55].

### 2.6. Density

The density of the solutions was measured with a 25 cm^3^ pycnometer (Everglass, Valparaiso, Chile) thermostated at 25 °C.

The partial specific volume ($\bar{v}$) of a polymer in solution is a measure of how much a unit mass of the polymer contributes to the total volume of the solution. It is not simply the inverse of the density of the pure polymer, since interactions between the polymer and the solvent can cause volume expansion or contraction. Prepare polymer solutions at different known concentrations (c) in a distilled water. It is important to dilute the solutions to minimize polymer–polymer interactions. Measure the solutions in a 25 mL pycnometer (IVA, Rosario, Argentina) at a controlled temperature [43].

(2) $$\delta =(v_{s}-\bar{v})/\rho_{0}$$

where $\bar{v}$ is the partial specific volume of the polymer, ρ is the density of the polymer solution, ρ_0_ is the density of the pure solvent and *v_s_* is the specific volume of the polymer solution (g/cm^3^).

### 2.7. Intrinsic Viscosity

Intrinsic viscosity is a measure of the contribution of a solute to the viscosity of a solution at infinite dilution, where the viscosity is measured in an Ubbelohde 1C viscometer (IVA, Rosario, Argentina). It is determined experimentally by measuring the relative viscosity (η_r_ = η ρ/η_0_ ρ_0_) or the specific viscosity (η_sp_) at different polymer concentrations (c) and extrapolating to c→0, where the specific viscosity is η_sp_ = (η − η_0_)/η_0_, and where η is the solution viscosity and η_0_ is the solvent viscosity, reduced viscosity is η_red_ = η_sp_/c and inherent viscosity is η_inh_ = ln(η_r_)/c. The intrinsic viscosity is obtained by extrapolating η_red_ to c = 0 (Huggins equation) or η_inh_ to c = 0 (Kraemer equation) [51].

Huggins equation:

η_sp_/c = [η] + k_H_ [η]^2^c (3)

Kraemer equation:

ln(η_r_)/c = [η] − k_K_ [η]^2^c (4)

where both extrapolations must converge to the same value of [η].

Other useful methods for verifying the above methods are the Kozicki–Kwang and Ishrak–Baranov methods. The first involves plotting η_sp_ as a function of concentration, the slope of which is the intrinsic viscosity. The second method involves plotting Ln η_r_ as a function of concentration, the slope of which gives the intrinsic viscosity [56].

The shape factor (ν_a/b_) gives us the shape of the molecule in solution, where the shape factor is related to the intrinsic viscosity and the specific volume of the solution, according to the following equation: [η] = ν_a/b_ v_sp_. For a rigid sphere, the Einstein shape factor is 2.5. For random coils or flexible chains (such as many polymers in a good solvent), the value of ν_a/b_ can be quite high than 2.5, reflecting the large hydrodynamic volume swept over the molecule in its rotational and translational motion.

#### 2.7.1. Molecular Weight Determination Using the Mark–Houwink Equation

The Mark–Houwink equation relates the intrinsic viscosity of a polymer to its molecular weight:

[η] = KM*^a^* (5)

where K and *a* are characteristic Mark–Houwink constants for a specific polymer-solvent system at a given temperature. These constants are obtained from calibrations with polymer samples of known molecular weight (Dextrans Serie T, Fluka, Berlin, Germany). Once [η] has been determined experimentally, the viscometric average molecular weight (M_v_) can be calculated using this equation [57].

The hydrodynamic radius of a macromolecule in solution can be estimated from the intrinsic viscosity using the modified Einstein–Stokes equation or the Flory–Fox relationship.

[η] = Φ (R_H_^3^/M_v_) (6)

where Φ is the Flory number, a theoretical constant that represents the ratio of the hydrodynamic volume to the volume of the polymer chain. However, we can use this relationship in combination with the Stokes–Einstein relationship for non-spherical particles to calculate R_H_, while R_H_ is a measure of how the molecule moves through the fluid (hydrodynamic radius). For a polymer that behaves like a random coil, even if it is not spherical, these equations can be combined to obtain a relationship between R_H_ and [η] [58].

Substituting this relationship into the Flory–Fox equation, we obtain an expression linking [η] and R_H_—[η] = Φ γ^3^ R_H_^3^/M—where γ is a theoretical constant that depends on the polymer conformation. For an ideal random coil, γ is approximately 1.5–1.6. From this equation, we can solve for R_H_ to calculate it from the intrinsic dry viscosity and molecular weight: R_H_ = (Φ γ^3^/[η] M)^1/3^.

The Flory number (Φ), often with a theoretical value of 2.5 × 10^23^ mol^−1^ for theta conditions, or an empirical value for the polymer–solvent system, is used [59].

This approach assumes that the polysaccharide can be modeled as a random coil. If the polysaccharide has a very rigid conformation, such as a rod or an extended helix, this relationship no longer holds. In those cases, specific hydrodynamic models for rods or ellipsoids would be used, which relate intrinsic viscosity to axial dimensions (length, diameter) and molecular weight.

#### 2.7.2. Hydration Value

The hydration value (δ), or solvation volume, represents the amount of solvent associated with a macromolecule. It can be estimated from the intrinsic viscosity by assuming that the hydrodynamic volume is composed of the dry volume of the polymer and the volume of the bound solvent.

(7) $$\left[\eta ]=\nu_{a/b}\;(\bar{v}+\delta \;v_{1}{}^{\circ}\right)$$

where $\bar{v}$ is the partial specific volume of the polymer (volume per unit mass of dry polymer), v_1_° solvent partial specific volume and δ is the hydration value (grams of solvent per gram of polymer) [60,61,62].

If a polysaccharide is not spherical, this suggests that it does not behave as a perfectly rigid sphere, which is in fact the most common behavior for polysaccharides in solution. Polysaccharides can adopt a variety of conformations, from more or less flexible random coils to more rigid chains or even branched structures or aggregates, depending on their chemical structure, molecular weight, and solvent conditions (temperature, ionic strength, pH).

### 2.8. Refractive Index and Optical Activity

The refractive index measurement is performed with a Schmitch-Haensch DUR (Berlin, Germany) thermostat set to 25 °C [63]. Optical activity was measured with a Carl Zeiss polarimeter, Berlin, Germany [63].

### 2.9. Surface Tension

The surface tension of the solutions was measured with a Du Noüy tensiometer (Brookfield, Middleboro, MA, USA) thermostated at 25 °C [43].

## 3. Results and Discussion

### 3.1. Proximal Analysis, Sugar Composition and Antioxidant Activity Assays

The process for obtaining *Neltuma flexuosa* exudate gum (NFEG) proved exceptionally efficient, achieving a high yield of 92 ± 3% wt. This is highly significant, as it indicates high productivity and potential industrial viability for the exploitation of this forest resource. Such a high yield minimizes raw material waste and reduces extraction costs at an industrial level, positioning NFEG as a highly available hydrocolloid.

Proximate analysis revealed a chemical composition characteristic of a gum exudate. The main component is the carbohydrate fraction at 89.9 ± 2% wt., which unequivocally confirms the polysaccharide nature of the gum. It is important to note that the protein content is 3.2 ± 0.11% wt. Contrary to what might be interpreted as an impurity, in Acacia and Prosopis/Neltuma gums, this protein fraction, covalently bound to the polysaccharide backbone, is functionally critical. This arabinogalactan–protein structure is responsible for the gum’s emulsifying and stabilizing properties, and its low concentration and high activity are what distinguish the best natural gums. The low ash (2.1 ± 0.56% wt.) and moisture (3.9 ± 0.78% wt.) content indicates an efficient purification process and excellent storage stability, respectively, since a low moisture content prevents microbial degradation. The absence of detectable fats reinforces its suitability as an additive in food or pharmaceutical formulations, where lipid oxidation is a critical factor.

These proximate values differ from those reported for *Neltuma flexuosa* seeds (which contain galactomannans), highlighting the distinct chemical nature of the gum exudates and the seed polysaccharides [24]. However, the composition of NFEG exhibits a marked similarity to the exudates of other Neltuma species (mesquite gum) and, crucially, to the exudates of Acacia species (arabic gum) [64]. This compositional convergence suggests a structural backbone and functionality very similar to arabic gum, the gold standard in the industry, validating the hypothesis that NFEG may be a high-value functional substitute.

Acid hydrolysis of NFEG allowed for the determination of its monosaccharide composition, an essential step in elucidating the polysaccharide’s molecular structure. The combined predominance of arabinose and galactose (approximately 75 ± 3.1% wt. of the total) unambiguously classifies NFEG as a polysaccharide belonging to the arabinogalactan family. It could be written that NFEG is a polyarabinogalactan. This structure is characterized by a backbone of D-galactose (42.2 ± 1.3% wt.) units and extensive branching via side chains containing L-arabinose (32.5 ± 1.3% wt.), which gives it a highly compact three-dimensional shape in aqueous solution. This random coil morphology is responsible for its characteristic low viscosity at high concentrations, a property shared with arabic gum and essential for applications requiring stability without excessive viscosity increase (see below), such as in beverages or emulsions.

A key structural aspect is the significant presence of uronic acids, totaling 11.6 ± 0.4% (8.5 ± 0.2% wt. methylglucuronic and 3.1 ± 0.1% wt. glucuronic). These carboxylic functional groups give NFEG a strong polyelectrolytic character. The negative charge density in the molecule is fundamental to its functional properties: it facilitates hydration, increases water solubility, and, most importantly, promotes electrostatic interaction with protein or lipid surfaces at water/oil interfaces, enhancing its stabilizing and emulsifying capabilities. The rhamnose content (3.2 ± 0.5%) is typical of arabinogalactans from exudates, often forming part of the main chain or linkage units.

Finally, the monosaccharide composition of NFEG underscores its striking similarity to both mesquite gum [8,10,16,17,18,24,37] (primarily from Neltuma) and arabic gum (*Acacia senegal* or *Acacia seyal*) [41,64]. This analogy in the constituent monomers and their relative proportions provides the chemical basis for NFEG to be considered and evaluated as a direct functional substitute for arabic gum [17,41,64,65,66,67] or mesquite gum [68], offering a valuable alternative derived from local natural resources.

The evaluation of the antioxidant activity of *Neltuma flexuosa* exudate gum (NFEG) by means of the DPPH Scavenging Activity (9.8 meq/g of gallic acid) and Reducing Power (5.1 meq/g of gallic acid) assays, together with the Total Polyphenols value (4.37 meq/g of gallic acid), reveals an intrinsic bioactive potential in the gum. These results suggest that NFEG is not only a structural hydrocolloid (thickener and emulsifier), but also carries compounds with the ability to neutralize free radicals and reduce oxidizing species, opening avenues for its use in functional foods and nutraceuticals. Although antioxidant activity is classically attributed to polyphenols (confirmed by the value of 4.37 meq/g of gallic acid), the polysaccharide structure of NFEG itself contributes to this effect. Arabinogalactans, and in particular the uronic acids present in NFEG (11.6% wt. glucuronic and methylglucuronic acid), are capable of chelating metal ions (Fe^2+^, Cu^2+^). This chelation is a secondary antioxidant mechanism, since by immobilizing transition metal ions, the polysaccharide inhibits the formation of highly reactive free radicals (such as hydroxyl radicals) through reactions like Fenton’s reaction. Furthermore, the arabinose- and galactose-rich branches can provide numerous free hydroxyl (-OH) groups that also act as hydrogen donation sites for radical deactivation (such as DPPH). Therefore, the high yield and sugar composition of NFEG not only define its rheological properties but also act synergistically with its low polyphenol content, validating its potential as a dual-functional additive with both water-repellent and bioactive properties [26,69,70,71].

### 3.2. Fourier Transform Infrared Spectroscopy

**Figure 1:**
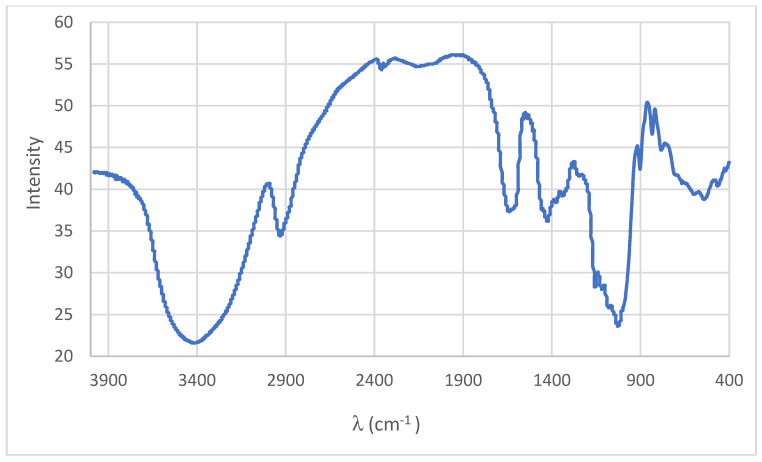
FTIR of *Neltuma flexuosa* exudate gum.

Figure 1 shows a Fourier Transform Infrared (FTIR) transmittance spectrum of a *Neltuma flexuosa* exudate gum (NFEG). Analysis of this spectrum allows us to identify the functional groups present in the sample, which in turn provides information about the gum’s chemical composition.

The broad and intense band centered around 3300–3400 cm^−1^ is characteristic of stretching vibrations of hydroxyl (-OH) groups. This is expected in polysaccharides, which are the main components of vegetable gums. The band width suggests the presence of inter- and intramolecular hydrogen bonds, common in polymeric structures with multiple hydroxyl groups [72].

The flattened and less intense bands around 2900–2950 cm^−1^ correspond to the C-H stretching vibrations of methyl (-CH_3_) and methylene (-CH_2_) groups. The presence of these bands indicates the organic nature of the material and is consistent with the structure of carbohydrates containing CH_2_OH and CHOH groups [72].

The polysaccharide fingerprint region (1800–900 cm^−1^) is crucial for polysaccharide identification, as it contains bands associated with the C=O stretching (if present), C–O, C–C, and O–H and C–H bending vibrations of the pyran and furan rings, as well as glycosidic bonds and pyranose and furanose structures, confirming the nature of arabinogalactan [64].

Although not the most prominent in this spectrum, a small absorption around 1730 cm^−1^ could indicate the presence of uronic acid ester groups (C=O of esterified -COOH) or fatty acid esters attached to the polysaccharide chain. Neltuma gums often contain uronic acids such as glucuronic or galacturonic acid [73,74].

A band around 1600–1650 cm^−1^ can be attributed to the bending vibration of adsorbed water [75], which is common in hydrophilic samples such as gums. There may also be contributions from the asymmetric stretching vibrations of carboxylate groups (-COO^−^) if the uronic acids are in deprotonated form [76].

The bands around 1420 cm^−1^ and 1370 cm^−1^ are typical of the C–H bending vibrations of methylene and methyl groups, respectively, and are consistent with the structure of carbohydrates [72].

The 1200–900 cm^−1^ region (anomeric and polysaccharide region). This is the most characteristic region for polysaccharides. The multiple intense bands in this region (especially between 1000 and 1100 cm^−1^) are attributed to the C-O stretching vibrations of the pyranose and furan rings, as well as to the glycosidic (C-O-C) bonds linking the monosaccharide units [77]. The shape and number of these bands are highly specific to the configuration and composition of the constituent monosaccharides (e.g., glucose, galactose, arabinose, xylose, rhamnose, etc.) and the types of glycosidic bonds (e.g., α-(1 → 4), β-(1 → 6)) [65]. *Neltuma flexuosa* exudate gums are complex polysaccharides containing a variety of monosaccharides and branched structures, which explains the complexity observed in this region of the spectrum [73,74]. Bands in the 900–750 cm^−1^ region (not very clear in the figure, but present in the shorter wavelength region) may indicate anomericity of the sugars (e.g., α- or β-configuration). For example, bands around 890 cm^−1^ are associated with β-glycosidic units, while those around 840 cm^−1^ may indicate α-glycosidic units [78,79].

The FTIR spectrum of *Neltuma flexuosa* exudate gum is consistent with the polysaccharide nature of the sample. The broad O-H bands, the C-H bands, and the complex polysaccharide fingerprint region (1200–900 cm^−1^) are characteristic of natural gums. The presence and shape of the bands in the 1730 cm^−1^ region (if significant) and subtle variations in the fingerprint region can provide further details about the presence of carboxyl groups and the specific composition of monosaccharides and glycosidic bonds, which is critical for understanding the functional properties of this gum [79,80,81].

### 3.3. X-Ray Diffraction

The XRD pattern exhibits a broad, diffuse band (also known as “amorphous halo”) centered approximately around 2θ ≈ 20°. The absence of sharp, well-defined diffraction peaks, which are characteristic of crystalline materials, is the most obvious indicator that the sample is predominantly amorphous [81,82].

In a crystalline material, the atoms or molecules are arranged in an ordered, repeating lattice, producing constructive interference of X-rays at specific angles (Bragg peaks). In contrast, in an amorphous material, the atomic or molecular arrangement is disordered at long range, although some short-range order may be present. This long-range disorder results in diffuse scattering of X-rays, creating the observed broad band [83,84].

**Figure 2:**
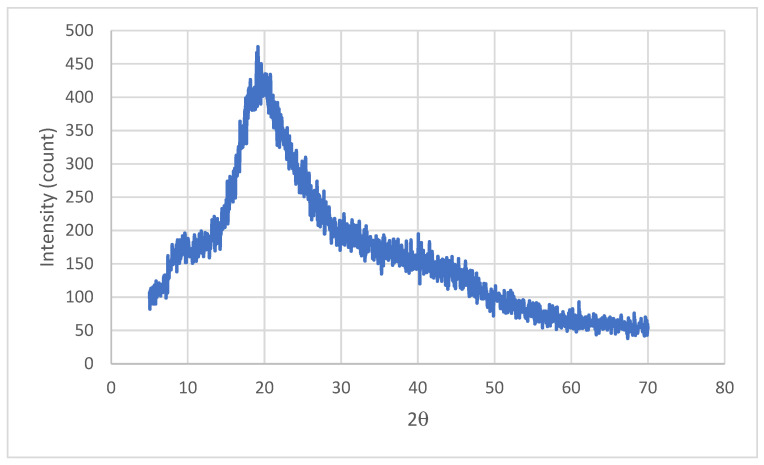
XRD of *Neltuma flexuosa* exudate gum.

Figure 2 shows an X-ray diffraction (XRD) pattern of a sample, which, considering the previous discussion about the ooze gum of *Neltuma flexuosa*, most likely corresponds to a polysaccharide or organic material with a high degree of structural disorder. It has a 2θ of 18.95°, d_-spacing_ of 2.37 ± 0.76 nm, and a crystallinity index of 32.72 ± 1.3%.

Exudate gums, such as those of *Neltuma flexuosa*, are complex, highly branched polysaccharides. Most natural polysaccharides, especially those that are not highly fibrous or cellulosic (such as cellulose, which has a well-defined crystalline structure), tend to exhibit amorphous or semi-crystalline behavior with a high percentage of amorphous phase in XRD [85,86]. The heterogeneous chemical structure and irregular branching of gums prevent the formation of a highly ordered crystal lattice, which is reflected in this amorphous pattern [87,88]. The broad band observed in the 2θ range of 15° to 25° (with a maximum around 20°) is common to many amorphous or low-crystallinity polysaccharides [85,86]. This band is associated with the average distances between polymer chains, or with repeating monosaccharide units that exhibit a certain local order [88].

The presented X-ray diffraction pattern is characteristic of a predominantly amorphous material, which is entirely consistent with the nature of a *Neltuma flexuosa* exudate gum, which is a complex, branched polysaccharide. This amorphous pattern is a direct consequence of the complexity and irregularity of its branching (given by the mixture of galactose, arabinose, rhamnose and uronic acid), which prevents an orderly and symmetrical molecular packing. The lack of sharp diffraction peaks indicates that the polymer chains lack a regular, repetitive long-range ordering. This amorphous behavior is important for understanding the physical and functional properties of the gum, such as its solubility, viscosity, and gel-forming ability, which are often influenced by the degree of crystallinity [88].

### 3.4. Differential Scanning Calorimetry

The glass transition temperature (T_g_) is the temperature at which the polymeric material (in this case, rubber) transitions from a hard, relatively brittle (glassy) state to a softer, rubbery (elastic) state. A value of T_g_ = 149.5 ± 1.2 °C is quite high for a natural rubber or biopolymer, suggesting a rigid molecular structure or the presence of strong intermolecular interactions (such as hydrogen bonds) that restrict the movement of the polymer chains at elevated temperatures. This high T_g_ value could indicate that the rubber has potential for applications requiring mechanical stability at elevated ambient temperatures.

The melting point (T_m_) is the temperature at which the crystalline regions (if any) of the polymer melt. For amorphous or semicrystalline polymers, the point between T_g_ and Tm defines their processing range. The difference between T_m_ and T_g_ is relatively small (approximately 21.6 ± 1.1 °C). This could indicate that the rubber is predominantly amorphous, that the crystalline regions are very small, or that the transition occurs within a narrow range. A melting point (T_m_) close to the glass transition temperature (T_g_) is typical of complex biopolymers that lack perfect crystallinity. DSC analysis of NFEG, with a residual moisture fraction (3.9% wt.), will show a broad endothermic transition at low temperatures, related to the loss of bound water and hydration of the hydroxyl and carboxyl groups with highly branched and amorphous arabinogalactan structure, rich in galactose and arabinose, will result in the absence of a defined crystalline melting peak.

The stability temperature (T_E_) refers to the maximum temperature at which a material maintains its structure and physical properties without undergoing significant chemical degradation (weight loss or measurable structural change). Thermal stability (T_E_) up to 250 ± 5 °C is excellent for a natural rubber or polysaccharide. This suggests that NFEG is thermally robust and can be used in formulation processes or applications involving heating to temperatures above 100 °C without immediate deterioration. The decomposition temperature (T_D_) marks the temperature at which thermal degradation is rapid and significant (often the point of maximum weight loss). This value of 315 ± 4 °C confirms the high thermal stability of the rubber. It is the upper limit for any application or processing. Above 315 ± 4 °C, the molecular structure of the rubber breaks down irreversibly. Finally, NFEG is a rubber with high thermal resistance for a natural material, giving it significant potential in various industrial or food applications where high-temperature stability is key.

The glass transition temperature (T_g_) of arabic gum and mesquite gum does not have a single, fixed value, as it depends largely on moisture content. Water acts as a plasticizer, meaning that higher humidity levels decrease the T_g_. Therefore, the values reported in the literature are associated with specific conditions. In the dry state, arabic gum can have a Tg of around 90 °C at low water content (0–40%), but this value can change dramatically with the addition of water and T_m_ of 145 °C [89].

Another 2022 study observed that incorporation of arabic gum into a powder increased the glass transition temperature up to 189.2 °C, suggesting that its T_g_ may be very high in the absence of moisture and in complex matrices, where the glass transition temperature is described to increase with the incorporation of tragacanth gum [90].

The T_g_ of mesquite gum varies with water content. There is no universally accepted value, as most studies focus on its behavior as a biopolymer in various applications. Typical values are 37.9 °C for mesquite gum under specific conditions. This value is significantly lower than those reported for arabic gum in the dry state, which could be due to differences in composition or measurement methodology [37].

Mesquite gum seed gum (MGS) varieties had a higher vitreous transition temperature (52.92 °C and 52 °C) than that found in other research (39 °C) [91,92,93]. For *Neltuma alba* exudate gum, T_g_ is 130 °C with activity water (a_w_) of 0.11 and arabic gum is 125 °C. The T_g_ 130 °C with a_w_ 0.11 and 50 °C for a_w_ 0.75 for prosopis gum [91,92,93].

### 3.5. Thermogravimetric Analysis and Differential Thermogravimetric Analysis

**Figure 3:**
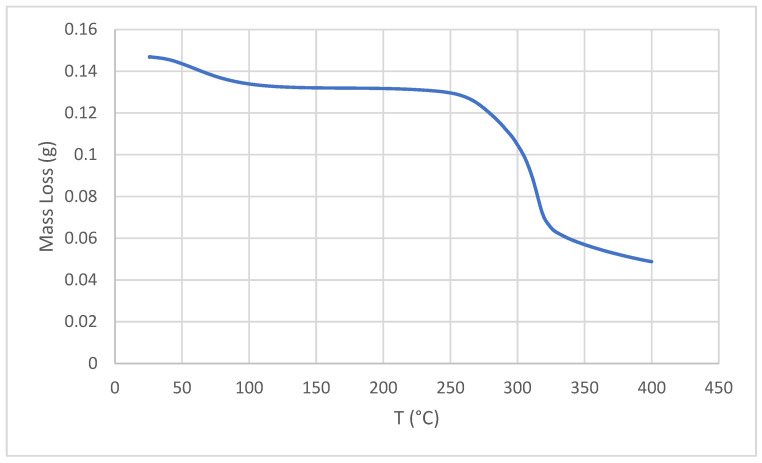
TGA of *Neltuma flexuosa* exudate gum.

**Figure 4:**
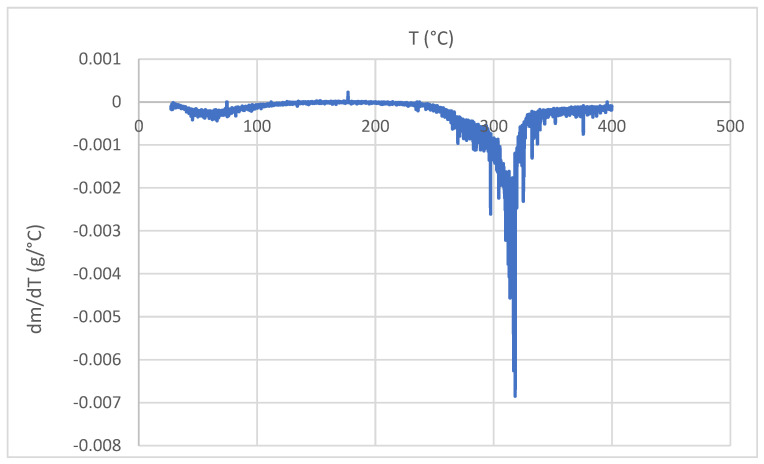
DTGA (dm/dT vs. T) of *Neltuma flexuosa* exudate gum.

Figure 3 and Figure 4 provided show the results of a thermogravimetric analysis (TGA) and a differential thermogravimetric analysis (DTGA) for the sample, which, in the context of previous discussions, is most likely an exudate gum from *Neltuma flexuosa* or a similar polysaccharide [94,95,96,97,98].

Figure 3 show thermal stability is up to 237.4 ± 3.1 °C, and it decomposes at 318 ± 4 °C. This plot present three stage:

The first stage of mass loss between 25 °C and 100 °C, a small but clear decrease in mass is observed from the initial mass loss up to approximately 100 °C. This first stage of mass loss, which appears to be around 2–3% (from 0.145 to 0.13), is generally attributed to the evaporation of moisture (water) adsorbed or physically associated with the material [83,84]. Natural gums, due to their hydrophilic nature and the presence of numerous hydroxyl groups, tend to retain a certain amount of water.

At the plateau or thermal stability (approx. 100–250 °C), after moisture loss, the TGA curve shows a region of relative thermal stability, where mass loss is minimal or zero, extending to approximately 250–260 °C. This indicates that the polymer is thermally stable in this temperature range before its main degradation begins [99,100].

In the second stage of mass loss starting at approximately 260 ± 6 °C, a significant and pronounced mass loss is observed. This stage corresponds to the thermal degradation or pyrolysis of the polysaccharide backbone [101]. For natural gums, this degradation involves the breaking of glycosidic bonds, depolymerization, and the formation of volatile compounds such as CO, CO_2_, water, and other decomposition products [101,102]. The point of highest degradation rate (most clearly visible in DTGA) indicates the temperature at which the most rapid decomposition occurs. The mass loss in this region is the most substantial, reflecting the decomposition of most of the organic material.

The third stage of mass loss beyond 350 ± 6 °C, the TGA curve continues to decrease, although at a slower rate. This could indicate the degradation of carbonaceous residues formed in the main pyrolysis stage, or the decomposition of more stable components that require higher temperatures [103]. If the sample were heated to even higher temperatures (e.g., 600 °C or 800 °C), the remaining mass at the end of the experiment would represent the carbonaceous residue or ash (inorganic material, if any) [104].

In Figure 4, a small valley (negative peak in dm/dT) is observed centered around approximately 70 ± 2 °C (or slightly lower, it is a subtle peak). This peak confirms the first stage of mass loss observed in TGA and corresponds to the maximum moisture evaporation rate [104,105]. The most prominent feature in the DTGA plot is a very sharp and narrow negative peak centered around approximately 310 to 320 °C. This is the main degradation peak and represents the temperature at which the sample undergoes the highest rate of mass loss due to decomposition of the polysaccharide backbone [106,107].

After the main peak, a tail or possible shoulders are observed, indicating that the degradation continues at higher temperatures, although at a decreasing rate. This could suggest the decomposition of components with different thermal stabilities or the secondary degradation of intermediates formed during the main pyrolysis [108]. In the case of complex polysaccharides such as gums, it is common to have multiple degradation stages due to structural heterogeneity [109].

TGA and DTGA analyses confirm the thermal stability of *Neltuma flexuosa* gum and its degradation behavior. The presence of moisture is an intrinsic characteristic of hydrophilic polysaccharides. The main thermal decomposition of arabinogalactan will occur at higher temperatures, generally between 250 °C and 350 °C. This stage corresponds to the degradation of glycosidic bonds and the fragmentation of the polysaccharide structure. These data are crucial for determining processing, storage, and application conditions for the gum where thermal stability is an important factor [110,111]. A high decomposition temperature is an advantage for its processing in food or materials that require drying or heating stages.

### 3.6. Intrinsic Viscosity and Density

Essentially, the relationship that highlights a polymer’s ability to increase the viscosity of a solvent (its intrinsic viscosity) is directly linked to how much space it occupies (specific volume) and how that space is distributed (form factor or conformation) in the solution. It is a tool used in polymer science to understand the size, shape, and interactions of macromolecules.

**Figure 5:**
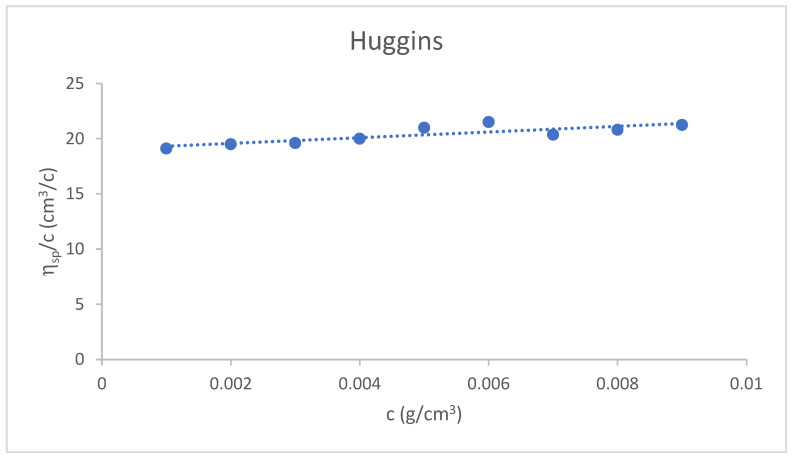
Intrinsic viscosity by Huggins method of *Neltuma flexuosa* exudate gum.

**Figure 6:**
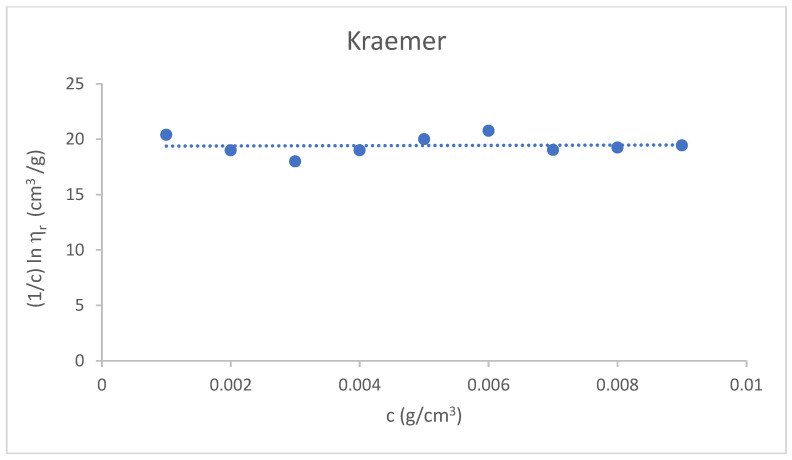
Intrinsic viscosity by Kraemer’s method of *Neltuma flexuosa* exudate gum.

Numerous studies focus on the rheological properties of gums obtained from different Neltuma species (see Figure 3, Figure 4, Figure 5 and Figure 6). These gum are polysaccharides and, when dissolved in water, form viscous solutions. Viscosity is a crucial property for their potential use in the food industry (as thickeners and stabilizers), pharmaceuticals, and other industrial applications.

Flow properties (Newtonian or non-Newtonian behavior, shear thinning), viscoelasticity (storage and loss moduli), and how these properties are affected by factors such as gum concentration, temperature, pH, and the presence of salts are investigated.

Examples of species studied include *Neltuma juliflora*, *Neltuma alba*, *Neltuma laevigata*, *Neltuma chilensis*, *Neltuma ruscifolia* “named vinal”, among others [18,112,113,114,115,116,117,118,119].

Some studies determine the intrinsic viscosity of Neltuma gum ex Prosopis gums, which provides information on the size and shape of macromolecules in solution. This is often related to the gum’s molecular weight and composition (e.g., the mannose/galactose ratio in galactomannans) [118].

It is common to find studies that compare the properties of mesquite gum with other known gums, such as arabic gum, to evaluate its potential as a substitute or alternative [6,113].

The viscosity of Neltuma extracts, particularly the mucilages of its fruits, has been evaluated for use as viscosity modulating agents in pharmaceutical formulations [18].

**Figure 7:**
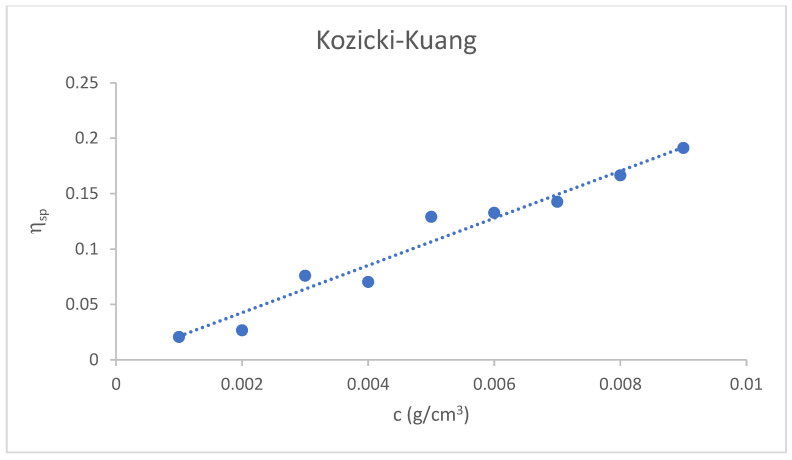
Intrinsic viscosity by Kozicki–Kuang method of *Neltuma flexuosa* exudate gum.

**Figure 8:**
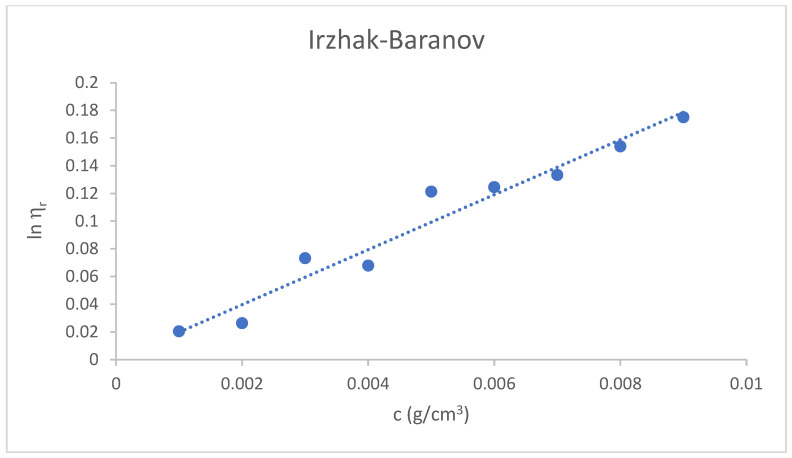
Intrinsic viscosity by Irhzak–Baranov method of *Neltuma flexuosa* exudate gum.

The methods of Kraemer (Figure 6), Kozicki–Kuang (Figure 7), and Irhzak–Baranov (Figure 8) are consistent with Huggins’ method (Figure 5). The data obtained are similar to those for arabic gum and mesquite gum, which are discussed below.

Intrinsic viscosity and molecular weight of gums, are: *N. glandulosa* 10 cm^3^/g with M 250,000 g/mol, *N. alba* 11 cm^3^/g with 460,000 g/mol, *N. valutina* 4.3 cm^3^/g with 83,000 g/mol, 12.6 cm^3^/g with 800,000 g/mol, N. juliflora 14 cm^3^/g with 310,000 g/mol, mesquite gum 13 cm^3^/g with 300,000 g/mol [39].

The molecular mass intervals for the different fractions were: *F*1 from 229,000 to 523,000 Da; *F*2 from 227,000 to 382,000 Da; and *F*3 from 18,100 to 347,000 Da. These molecular masses are substantially lower than the reported average molecular mass for whole MG obtained from *N. laevigata* which is about 212,000 Da [8,111,120].

Intrinsic viscosity of gum solutions in 0.1 M NaCl was estimated at 25 °C using a 0B size Ubbelohde capillary viscometer (Technical Glass Products). The viscosity average molecular weight (M_n_) of the mesquite gum samples was calculated using the obtained intrinsic viscosity values in the Mark–Houwink equation using k (0.147 and 0.151) and *a* (0.50 and 0.52) constants reported for mesquite and arabic gum, respectively [121]. In mesquite gum the reference [37] was 8.7–9.9 cm^3^/g with 350,000–453,000 g/mol. In *Neltuma alba* exudate gum the reference [113] was 17.7 cm^3^/g [122].

The intrinsic viscosity ([η]) is a fundamental parameter in the characterization of polymers, including polysaccharides such as Neltuma/Prosopis gums, because it provides information about the size and hydrodynamic shape of the macromolecules in solution and correlates with the average molecular weight of the polymer [60,111,123].

This study compares the rheological properties of mesquite gum with arabic gum and reports intrinsic viscosity values. It mentions that the intrinsic viscosity of mesquite gum ([η] ≈ 11 dL/g) was appreciably lower than that of arabic gum ([η] ≈ 19 dL/g), suggesting a more compact structure [7]. In reference [60], the intrinsic viscosity of arabic gum was 19.81 cm^3^/g and the molecular weight was 760,000 g/mol. Acacia caven gum presents an intrinsic viscosity of 46.05 cm^3^/g and the molecular weight is 1,060,000 g/mol [61]. The *Neltuma flexuosa* gum presents an intrinsic viscosity of 20.79 cm^3^/g [32].

This specific study on the seed gum of *Prosopis juliflora* (as distinct from the exudate) reports intrinsic viscosity values (e.g., 11.78 cm^3^/g in one sample) and calculated molecular weight. It is important to note that seed gums (galactomannans) typically have much higher intrinsic viscosities than exudates [124].

This study compares exuded gums from different *Neltuma* species and reports ranges of intrinsic viscosity (between 10.6 and 13.6 cm^3^/g), using these values to calculate the viscometric average molecular weight using the Mark–Houwink equation [125,126].

The viscosity of NFEG, as an arabinogalactan, is expected to be relatively low compared to other hydrocolloids such as xanthan gum or guar gum, even at high concentrations. This low viscosity is a direct consequence of its highly branched and compact molecular structure (random coil shape), which minimizes hydrodynamic drag in solution. Functionally, this is advantageous for stabilization or encapsulation applications where a high solids content is required without altering the final texture. The density of the solutions will increase linearly with the carbohydrate concentration (89.9% wt.) and is the key parameter for the accurate preparation of these solutions.

The data of Table 1 suggest that *Neltuma flexuosa* exudate gum in solution has a moderately high mean molar mass (M = 328,400 ± 11 g/mol). Adopts a random coil conformation with little or close to a theta-solvent expansion, as indicated by the Mark–Houwink exponent *a* = 0.55. This is typical of branched polymers. The hydrodynamic radius (R_H_ = 10.12 ± 0.4 nm) is relatively small for its molecular weight, confirming that the molecule is compact or highly branched in solution, similar to the arabinogalactan–protein structure of other exudate gums. The low intrinsic viscosity value [η] = 19.90 ± 0.5 cm^3^/g is consistent with its compact structure and the low value of the exponent *a*. These results are consistent with the characterization of many exudate plant gums, such as arabic gum [7,60], which often exhibit a branched and compact structure. The inclusion of δ and ϕ in the hydrodynamic analysis indicates that the study used a sophisticated (not simple) model to compensate for the highly branched and compact shape of the *Neltuma flexuosa* exudate gum. These parameters served as calibration and conversion constants to move from the measured viscous properties (such as [η]) to the actual physical dimensions of the hydrated molecule in solution (R_H_ and M), similar at arabic gum and mesquite gum [60].

#### Temperature Effect on Intrinsic Viscosity

**Figure 9:**
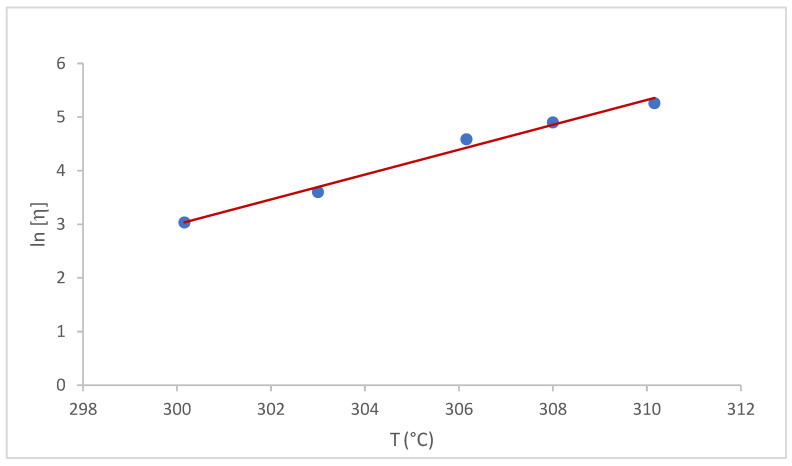
Flexibility chain of NFEG.

Figure 9 shows the natural logarithm of the intrinsic viscosity, ln[η], as a function of absolute temperature, T(K), for the gum *Neltuma flexuosa* (ex *Prosopis flexuosa*) in aqueous solution. This representation is crucial for understanding the flexibility or conformational behavior of the polymer as a function of temperature, a phenomenon governed by fundamental physicochemical principles.

- Figure 9 exhibits a clear linear trend with a positive slope, described by the regression line equation: ln[η] = 0.232x − 66.598.
- The slope is positive, meaning the intrinsic viscosity, [η], increases as the temperature, T, increases (since ln[η] also increases).
- The value of R^2^ = 0.9868 indicates an excellent fit of the experimental data to a linear model, suggesting that the mechanism relating [η] and T is consistent across the temperature range studied.

The intrinsic viscosity, [η], is a measure of the hydrodynamic volume a polymer molecule occupies in a solution and its ability to impede solvent flow. For a flexible polymer such as a rubber (a polysaccharide), [η] is directly related to its conformation (the shape and extension of the chain) in solution.

For most flexible polymers in athermal or “good” solvents (such as water for this gum), an increase in temperature results in an increase in intrinsic viscosity (the slope is positive). In this temperature range, water behaves as a better solvent for the gum. With increasing temperature: a—The kinetic energy increases, allowing water molecules to interact more effectively with the hydrophilic functional groups (mainly −OH) of the polysaccharide. The enthalpy of mixing (ΔH_mixing_) becomes less unfavorable or more favorable. b—The attractive forces between polymer segments are weakened or overcome by polymer-solvent interactions. c—The predominance of polymer–solvent interactions and the steric repulsion between segments (since the chain is “better solubilized”) force the polymer chain to expand and unwind. d—The chain changes from a more compact conformation (smaller hydrodynamic volume) to a more extended and random conformation (larger hydrodynamic volume). This process is entropically favorable (ΔS > 0) for the chain, since it explores a greater number of possible microstates. e—By occupying a larger hydrodynamic volume, the extended molecule offers greater resistance to the laminar flow of the solvent, which translates into an increase in intrinsic viscosity. f—The increase in temperature increases the rotational kinetic energy of the polymer chain segments (glycosidic bonds and internal bonds of the sugar rings). This facilitates the free rotation of the segments around the single bonds, increasing the local flexibility of the polymer. Molecular collisions are more frequent and energetic, which helps to break the aggregates or weak interactions (H-bonds, van der Waals) that stabilize the compact shape, promoting conformational relaxation and expansion. g—In the context of viscosity as a function of temperature, the slope (dln[η]/dT) is often used as a relative measure of “flexibility” or “conformational change” with temperature, where dln[η]/dT = 0.232 K^−1^ (see Figure 9). This positive value indicates that the hydrodynamic volume of *Neltuma flexuosa* gum is highly sensitive to temperature changes over the range studied. A high value suggests that the chain is relatively flexible and that polymer-solvent interactions improve markedly with increasing T, promoting significant expansion. Stiffer polymers or those in “lean solvent” conditions would show a less steep slope, or in some cases, a negative slope (if the polymer compacts with increasing temperature, e.g., due to a phase transition or aggregation) [58,60,61,62,126].

### 3.7. Refraction Index

**Figure 10:**
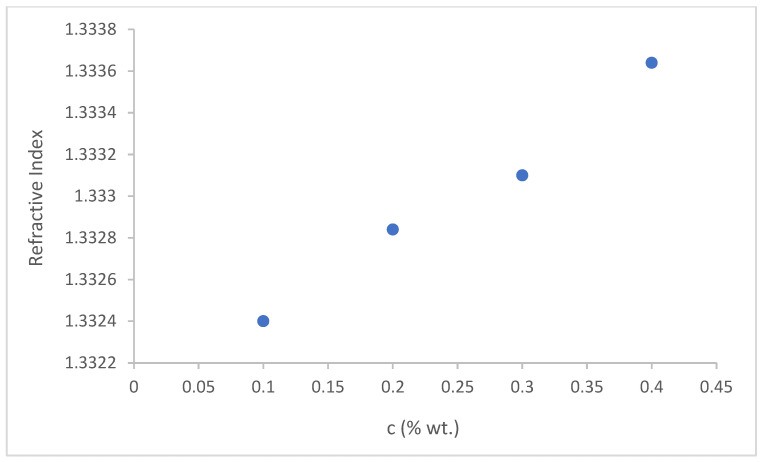
Refraction index of NFEG.

Figure 10 shows the relationship between the refractive index and concentration (% wt.) of a solution. Analysis of the graph indicates that the refractive index of the solution increases as the concentration of the dissolved substance increases. This behavior is typical and expected, since the addition of a solute to a solvent generally alters the optical properties of the solution, including its refractive index. The general trend is a directly proportional relationship, where an increase in concentration leads to an increase in the refractive index. This suggests that the substance under study contributes significantly to the optical density of the solution.

The refractive index is a useful tool for studying the hydration of macromolecules, albeit in an indirect and complex manner. There is no single “δ” value that can be directly measured with a refractometer, as the refractive index is a macroscopic property that reflects the combined effect of all the molecules in a solution [127].

The hydration of macromolecules refers to the water molecules that are either associated with their surface or trapped within their structure, forming a “hydration shell” or “solvation shell.” This water behaves differently than bulk water and is crucial to the stability and function of the macromolecule. The “hydration delta” (ΔH_hyd_, often referred to as the hydration enthalpy) refers to the energy released when one mole of ion or a macromolecule dissolves in a large amount of water. This value is not directly measured with a refractometer [128].

The refractive index of a solution containing macromolecules is affected by several factors, including: (1) The higher the concentration, the higher the refractive index. (2) Different components (amino acids, nitrogenous bases, monosaccharides, etc.) have different optical properties. (3) Changes in the structure of the macromolecule (e.g., conformational changes upon binding to another molecule) can alter the size and polarizability of its hydration shell. These subtle changes in the hydration shell modify the dielectric response of the solution and, therefore, its refractive index [129].

Measurement of the change in refractive index (n) has been used in research to study molecular interactions, such as the binding of calcium ions to proteins or the interaction between a ligand and an enzyme. These “n” changes, although small, can be quantified and are related to the conformational and hydration changes that occur during the interaction [130].

However, calculating the amount of water of hydration (in grams of water per gram of macromolecule) requires more complex models and equations that combine the refractive index with other properties, such as specific partial volume and molecular mass. There is no simple formula that allows the “delta hydration value” to be directly obtained with a general-purpose refractometer [131,132].

The refractive index is a fundamental property affected by the hydration of macromolecules, but it is primarily used in research studies to infer changes in the hydration shell, not to obtain a direct measurement of the “delta hydration value” [129,133].

In the context of macromolecules, there is no single equation that allows a “delta hydration value” to be directly calculated from a simple refraction measurement [134,135]. However, the hydration of macromolecules is studied using equations that relate the refractive index (a macroscopic property) to molecular properties [136,137,138].

One of the fundamental equations used for this is the Lorentz–Lorenz equation, which establishes a link between the refractive index and molecular polarizability, *α*′.

(8) $$\left(\frac{n^{2}-1}{n^{2}+2}\right)=1.33\;\pi \;N\;\alpha^{\prime }$$

Molar Refraction

(9) $$R_{M}=\left(\frac{n^{2}-1}{n^{2}+2}\right)\frac{M}{\rho }=\frac{N_{A}\alpha^{\prime }}{3\varepsilon_{0}}$$

where *R_M_* is the molar refraction, *α*′ is the property that reflects the size and polarizability of the molecules C^2^ m^2^/J, n is the refractive index of the solution, *M* is the molar mass of the substance, *ρ* is the density of the solution (g/m^3^) and *ε*_0_ = 8.8542 × 10^−12^ C^2^/J m, where the frequency of the refractive index measurement is the D line of sodium at 589.3 nm [136].

The hydration shell surrounding a macromolecule has different dielectric and polarizability properties than water. Therefore, when a macromolecule undergoes a conformational change (e.g., by binding to another molecule), changes occur in the size and structure of its hydration shell.

The term alpha polarizability (α) is a fundamental measure in physics and chemistry that quantifies the ease with which the electron cloud of an atom, molecule, or ion can be deformed or distorted by the presence of an external electric field [137].

Essentially, α describes the ability of a chemical species to be induced with a temporary dipole moment.

The polarizability α is crucial to understanding several phenomena: 1—Polarizability is the direct cause of London dispersion forces (a form of Van der Waals forces). A high α implies stronger dispersion forces, which translates into higher boiling and melting points. 2—The α at optical frequencies is directly related to the refractive index of a material. 3—The molecular α is related to the dielectric constant (κ or *ε_r_*) and the electrical susceptibility (χ_e_) of the medium [138].

These changes manifest as a change in the refractive index of the solution (n). Researchers use theoretical models, based on the Lorentz–Lorenz equation, to relate this measured change in refractive index to variations in the polarizability of the macromolecule and its solvation shell.

The Clausius–Massotti equation,

(10) $$\left(\frac{n^{2}-1}{n^{2}+2}\right)=\frac{\left(\varepsilon_{r}-1\right)}{\left(\varepsilon_{r}+2\right)}=\frac{N_{A}\alpha \rho }{3\varepsilon_{0}M}$$

Solution permittivity or dielectric constant

(11) $$\varepsilon_{r}=\left(\frac{\frac{2N_{A}\rho \alpha }{3\varepsilon_{0}M}+1}{1-\frac{N_{A}\rho \alpha }{3\varepsilon_{0}M}}\right)≅\;n^{2\;}\;$$

The relationship *ε_r_* ≅ *n*^2^ is fundamental (Maxwell’s relationship) and applies to the dielectric constant at high frequencies (*ε*_∞_). In the field of polysaccharide and biopolymer solutions, this relationship is often used in scientific articles, not as a novel finding, but as a basic principle for interpreting dielectric and optical properties.

**Figure 11:**
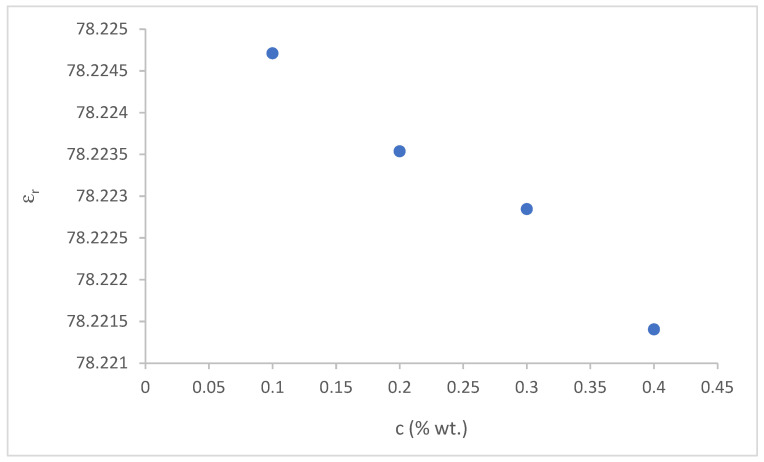
Permittivity of NFEG aqueous solution.

The provided Figure 11 displays the relative dielectric permittivity (ε*_r_*) in function of concentration (c % wt.) for a dilute aqueous solution of a polysaccharide derived from the NFEG.

The solution permittivity (ε*_r_*) values are a measure of a substance’s ability to store electrical energy in an electric field and are presented on a very narrow scale, from approximately 78.221 to 78.225 (see Figure 11). The range of values suggests that the predominant solvent is water, whose permittivity at room temperature is high (approximately 78–80). The concentration varies from 0.1 to 0.4% wt., confirming that this is a dilute solution. This decrease in *ε_r_* with increasing solute concentration is typical behavior in aqueous solutions of nonionic or weakly ionic solutes, such as polysaccharides.

The observed effect is known as dielectric depression or negative solvation effect on solvent permittivity: 1—The high ε*_r_* value of water is due to the high polarity of its molecules and their ability to form a hydrogen bonding network that allows easy reorientation under an electric field. 2—When the polysaccharide (solute) dissolves in water (solvent), its functional groups (mainly -OH) interact strongly with the surrounding water molecules, forming a solvation (or hydration) water shell around the polymer. 3—The water molecules trapped in this solvation shell are now preferentially oriented or immobilized (with their rotational and translational freedom restricted) by the solute. This immobilization significantly reduces their ability to freely reorient in response to an external electric field. 4—The portion of water that has lost its rotational freedom contributes little or nothing to the total dielectric permittivity of the solution. Since the polysaccharide itself has a much lower intrinsic *ε_r_* than water, the net result is a decrease in the relative permittivity of the total solution as the solute concentration increases. The magnitude of the dielectric depression (the slope of the curve) is directly related to the amount of solvation water immobilized per unit concentration of the polysaccharide [63].

Figure 11 confirms that the polysaccharide from *Neltuma flexuosa* exudate gum acts as a dielectric permittivity suppressor in aqueous solution. This property is not only an intrinsic feature of the system but also provides valuable information about the water-polysaccharide interaction at the molecular level, suggesting a strong degree of hydration or solvation that restricts the mobility of water molecules. The measurement is highly precise, as evidenced by the narrow range of the *ε_r_* axis, which is crucial for detecting these subtle changes in such dilute solutions.

**Figure 12:**
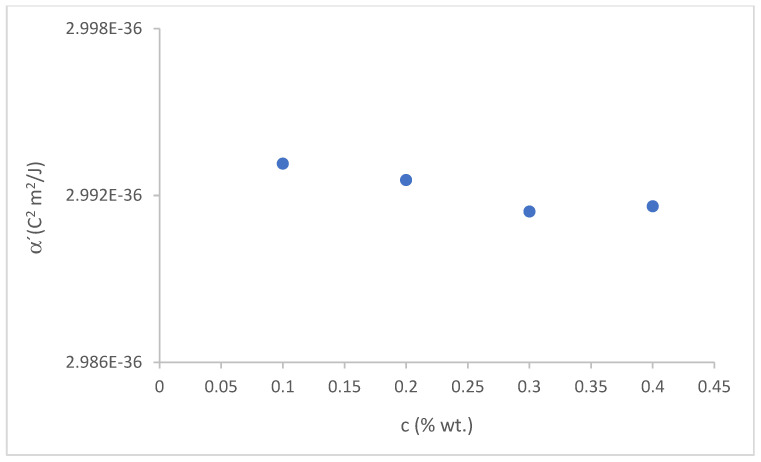
Polarizability of NFEG.

A decrease in polarizability with increasing concentration suggests that intermolecular interactions are increasingly important (see Figure 12). At higher concentrations, molecules are closer together and may begin to “interfere” or “shield” each other’s individual ability to polarize. This can be due to an aggregation/association phenomenon. That is, if molecules form aggregates (dimerization, micelles, etc.), their electronic structure may be more rigid or less available to distortion, reducing the effective polarizability per molecule. If the sample is a solution, changing the solute concentration can alter the solvent structure or the solute–solvent interactions in a way that restricts electron mobility. Increasing polarizability at 0.4% wt. causes a slight increase that could indicate the onset of a phase change or a change in the interaction mechanism. For example, a molecular reorientation or the formation of a different aggregate structure that, paradoxically, allows for slightly greater polarization than the previous structure.

The refractive index of NFEG solutions increases with concentration due to the high carbohydrate content (89.9% wt.), which enhances the optical density of the medium. This property is useful for quality control. The dielectric permittivity (*ε_r_*) in aqueous solutions is closely linked to the hydration and ionic character of the gum (11.6% wt. uronic acids). The presence of carboxylate groups and the dipoles of hydroxyl groups are expected to induce an increase in permittivity at certain analysis frequencies (dielectric dispersion), reflecting the interactions between water and the polyelectrolyte, which are crucial for its functionality as a stabilizer in emulsions.

#### Optic Activity

**Figure 13:**
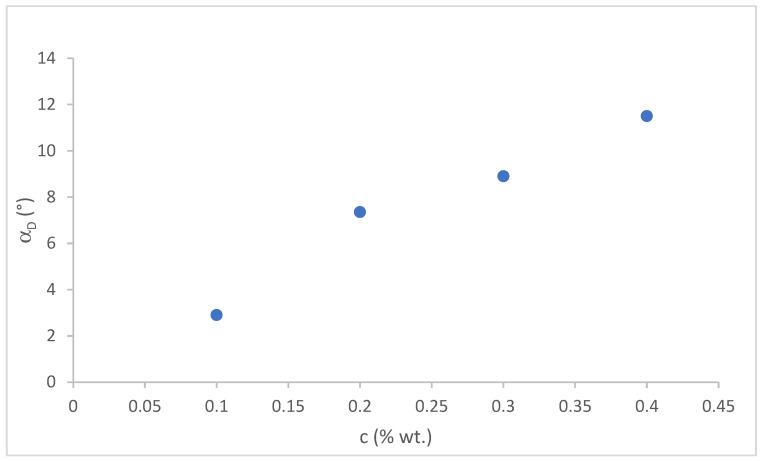
Optical activity of NFEG.

Looking at Figure 13, the optical activity of *Neltuma flexuosa* exudate gum can be evaluated as a function of its concentration. The graph shows the angle of optical rotation (α_D_) versus the concentration (c) (% wt.).

The data points indicate that as the gum concentration increases, the angle of optical rotation also increases, demonstrating a directly proportional relationship between the two variables. This suggests that *Neltuma flexuosa* exudate gum is optically active and dextrorotatory; that is, it has the ability to rotate the plane of polarized light [64].

This linear upward trend is characteristic of optically active substances in solution, where the degree of rotation is directly proportional to the solute concentration and the optical path length.

The optical activity of NFEG is measured as the specific rotation (α_D_) of polarized light, reflecting its chiral nature (presence of asymmetric centers in the sugars galactose, arabinose, and rhamnose). The specific rotation will be a positive or dextrorotatory value, characteristic of arabinogalactan and galactomannan structures. This value is an important fingerprint for the identification and purity control of NFEG, as it depends on the spatial configuration of the monosaccharides (e.g., D-galactose and L-arabinose) and the specific glycosidic bonds present in the branched structure.

### 3.8. Surface Tension

**Figure 14:**
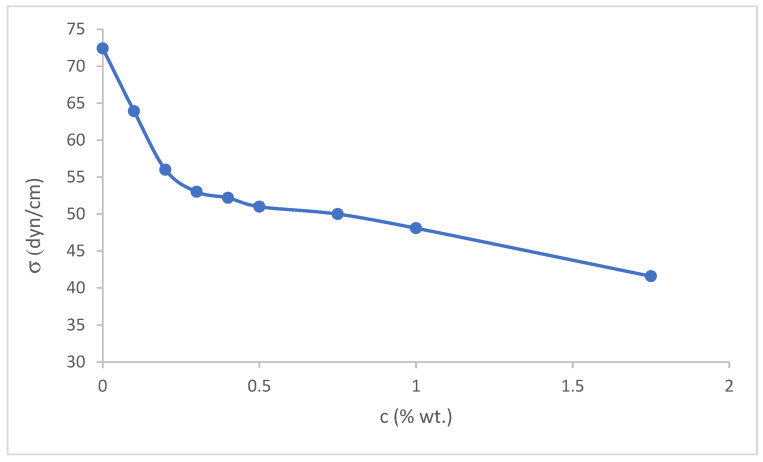
Surface tension of NFEG.

Figure 14 shows the relationship between surface tension (σ) in dyn/cm and concentration (c) (% wt.) of *Neltuma flexuosa* exudate gum. The plot reveals that the addition of NFEG to a solution decreases its surface tension. This behavior is typical of surfactants or substances with surface-active properties, which tend to accumulate at the air–liquid (or liquid–liquid) interface and reduce the surface free energy.

Looking at the data points, a pronounced decrease in surface tension can be seen at low initial concentrations, followed by a more gradual decrease as the concentration increases. The curve suggests the formation of a monolayer or saturation of the interface at higher concentrations, where adding more gum no longer produces such a drastic reduction in surface tension. This behavior indicates that *Neltuma flexuosa* exudate gum possesses surfactant properties, which is relevant for its potential use as an emulsifying or stabilizing agent in various industrial applications [44,64].

The surface tension of aqueous NFEG solutions is a key property directly related to their ability to act as an emulsifying and foam-stabilizing agent. The 3.2% protein content, bound to the polysaccharide fraction, acts as a natural surfactant. This glycoprotein tends to adsorb at the water/air or water/oil interface, reducing interfacial tension and forming a viscoelastic film that stabilizes droplets. Therefore, NFEG solutions are expected to exhibit a significant reduction in surface tension compared to pure water, a functional characteristic that again resembles that of arabic gum.

## 4. Conclusions

The comprehensive physicochemical characterization of the exudate of *Neltuma flexuosa* (ex *Prosopis flexuosa*) has solidified its position as a natural hydroxycolloid with an exceptionally valuable property profile for diverse industrial applications. Results obtained using a battery of analytical techniques confirm the central hypothesis that this gum is a viable and comparable alternative to arabic gum, highlighting its complex composition, favorable rheological behavior, and remarkable stability. Proximate analysis and sugar composition identified NFEG as an arabinogalactan–protein (89.9% wt. of carbohydrates, 3.2% wt. of protein), with a high proportion of uronic acids (11.6% wt.) that confer a crucial polyelectrolytic character. It could be written that NFEG is a polyarabinogalactan. This structure is remarkably similar to arabic gum, validating NFEG as a functional substitute with emulsifying and stabilizing capacity. In addition to its structural properties, NFEG exhibits intrinsic bioactive potential, displaying significant antioxidant activity (DPPH: 9.8 meq/g, polyphenols: 4.37 meq/g). This dual functionality, both structural and bioactive, underscores the importance of valuing this native forest resource as a high-performance, local, and sustainable alternative in the food and pharmaceutical industries. At the structural and compositional level, Fourier Transform Infrared Spectroscopy (FTIR) analysis unambiguously demonstrated the polysaccharide nature of the gum. The presence of a broad, intense band between 3300 and 3400 cm^−1^ is characteristic of O-H stretching vibrations, while the fingerprint region of 1800–900 cm^−1^ is consistent with glycosidic linkages and carbohydrate rings. The possible presence of esterified uronic acids (C=O vibration around 1730 cm^−1^) suggests the presence of functional groups that contribute to its polyelectrolytic character and, therefore, its functionality. X-ray diffraction (XRD) complements this structural profile by revealing that *Neltuma flexuosa* exudate gum is a predominantly amorphous material with a high degree of structural disorder. The lack of sharp peaks and the presence of a diffuse band around 2θ of 18.95° (with a reported crystallinity index of 32.72%) reflect the complexity and irregular branching of its polysaccharide chains, a typical feature of exudative gums. This amorphous behavior is key to its high solubility and ability to form viscous solutions.

Regarding its properties in solution, the intrinsic viscosity confirms the remarkable hydrodynamic potential of the gum. The [η] values for NFEG are 19.90 to 22 cm^3^/g comparable to that of arabic gum (19.81 cm^3^/g), projecting similar performance as a thickening agent. The high chain flexibility (0.2251 K^−1^) and the v_a/b_ shape factor of 3.13 indicate that the macromolecule, although resembling a sphere, exhibits a flexible conformation that expands as the temperature increases, suggesting that water behaves as a “better solvent” under these conditions. Optical and thermal properties reinforce the characterization. Optical activity data demonstrate a directly proportional relationship between concentration and rotation angle, confirming that the gum is optically active and dextrorotatory.

The refractive index also exhibits an increase with concentration. Meanwhile, thermal analysis by TGA-DTGA reveals considerable thermal stability, with the onset of decomposition in the 237.4 °C region and a main peak. The degradation of the polysaccharide backbone at around 318 °C is fundamental data for its processing and application in products requiring heat treatment. Finally, surface tension provides one of the most relevant functional findings. *Neltuma flexuosa* exudate gum reduces the surface tension of the solution, an unequivocal behavior of a substance with surfactant properties. This effect, which shows saturation of the interface at high concentrations, is crucial, as it gives the exudate the ability to act as an emulsifying and stabilizing agent in various industrial matrices. The convergence of structural, hydrodynamic, and functional data firmly establishes that NFEG is a promising arabinogalactan–protein. The physicochemical evidence obtained, which highlights its high flexibility, similarity to arabic gum, and surfactant properties, validates the need to continue research toward standardizing its processing and exploring its modifications. The valorization of this native resource not only offers a sustainable alternative but also present local alternatives to imported hydrocolloids, but also promotes the intelligent use and conservation of forest species in arid regions.

*Neltuma flexuosa* exudate gum is not simply another polymer, but a strategic bioresource that encapsulates the promise of green chemistry and regional biotechnology. The physicochemical evidence compiled here not only validates its potential as a high-performance functional ingredient, but also issues a call to action for the protection, valorization, and sustained investment in natural heritage. Only through continuous research and a commitment to innovation will it be possible to transform this native exudate into an engine of sustainable development and a real alternative to petrochemical-based polymers, reaffirming the fundamental role of biodiversity in solving the technological challenges of the 21st century.

## Figures and Tables

**Table 1:** Intrinsic viscosity and hydrodynamic data by Huggins method of *Neltuma flexuosa* exudate gum.

[η] (cm^3^/g)	k (cm^3^/g)	*a*	M_v_ (g/mol)	δ (g/g)	ν_a/b_	R_H_ (nm)	Φ (mol^−1^)
19.90 ± 0.5	0.0184	0.55	328,400 ± 11	0.45 ± 0.02	3.13 ± 0.09	10.12 ± 0.4	4.29 ± 0.11e^−21^

## Data Availability

The original contributions presented in this study are included in the article. Further inquiries can be directed to the corresponding authors.
